# The Impact of Underlying Plaque Characteristics Following the Third‐Generation Resorbable Magnesium Scaffold Implantation: An Intravascular OCT Assessment up to 12‐Months

**DOI:** 10.1002/ccd.31486

**Published:** 2025-03-16

**Authors:** Alp Aytekin, Masaru Seguchi, Erion Xhepa, Michael Haude, Adrian Wlodarczak, René J van der Schaaf, Jan Torzewski, Hector M. Garcia‐Garcia, Ron Waksman, Michael Joner

**Affiliations:** ^1^ Klinik für Herz‐ und Kreislauferkrankungen, Deutsches Herzzentrum München Munich Germany; ^2^ Medical Clinic I, Rheinland Klinikum Neuss GmbH, Lukaskrankenhaus Neuss Germany; ^3^ Department of Cardiology Miedziowe Centrum Zdrowia SA Lubin Poland; ^4^ Faculty of Medicine Wroclaw University of Science and Technology Wroclaw Poland; ^5^ Department of Interventional Cardiology OLVG Amsterdam The Netherlands; ^6^ Cardiovascular Center Oberallgäu‐Kempten Kempten Germany; ^7^ Interventional Cardiology, MedStar Washington Hospital Center Washington DC USA

**Keywords:** bioresorbable magnesium scaffold, coronary artery disease, optical coherence tomography, percutaneous coronary intervention, plaque characteristics

## Abstract

**Background:**

Third‐generation resorbable magnesium scaffold (RMS) was developed with stronger mechanical properties and thinner struts compared to its predecessor. This study aimed to assess the influence of the OCT‐derived underlying plaque characteristics on in‐scaffold late lumen loss (LLL) in patients treated with RMS up to 12‐months.

**Methods:**

Patients enrolled in the BIOMAG‐I trial and who underwent OCT imaging before and after the index procedure were included in the current analysis. The acquired intravascular imaging data were evaluated to assess the presence of fibrous, calcific, or lipidic lesions. We calculated the proportions of each plaque feature per pullback and assessed their correlation with LLL obtained at 6‐ and 12‐months follow‐up. In addition, we investigated the potential impact of scaffold edge dissection and strut malapposition on in‐scaffold LLL.

**Results:**

Eighty‐four patients and 84 lesions were evaluated in the current analysis. There was no significant correlation between the underlying plaque characteristic and in‐scaffold LLL at 6‐months (*p* = 0.79 for fibrous, *p* = 0.88 for calcific, *p* = 0.67 for lipid lesions). This trend was similar at 12 months follow‐up (*p* = 0.56 for fibrous, *p* = 0.75 for calcific, *p* = 0.69 for lipid lesions). The presence or absence of edge dissection did not influence the degree of in‐scaffold ‐ (*p* = 0.51 at 6‐months, *p* = 0.68 at 12‐months follow‐up) or in‐segment LLL (*p* = 0.88 at 6‐months, *p* = 0.70 at 12‐months follow‐up).

**Conclusion:**

The underlying plaque characteristics, edge dissection or strut malapposition had no significant impact on in‐scaffold LLL following DREAMS 3 G implantation up to 12 months. This suggests better device performance irrespective of the underlying plaque characteristics.

**Trial Registration:**

ClinicalTrials.gov ID: NCT04157153.

## 1 Introduction

Second‐generation resorbable magnesium scaffold (RMS) Magmaris showed favorable outcomes in clinical trials, especially regarding the incidence of scaffold thrombosis [1]. The earlier studies reported a higher occurrence of scaffold recoil with this RMS owing to vessel constriction. This finding was shown to be more prominent in presence of underlying fibrous plaques rather than calcific and/or lipid lesions [2] and would suggest that sufficient radial force along with longer scaffolding time may improve the device performance.

DREAMS 3 G was developed with stronger mechanical properties despite thinner struts by modifying the magnesium alloy of its past iterations [3]. The first‐in‐man trial BIOTRONIK—Safety and Clinical Performance of the Sirolimus‐Eluting Resorbable Coronary Magnesium Scaffold System in the Treatment of Subjects With de Novo Lesions in Native Coronary Arteries (BIOMAG‐I) demonstrated promising results for the novel sirolimus‐eluting RMS with respect to clinical and angiographic outcomes at 6 and 12‐months follow‐up [4, 5]. Still, potential impact of the underlying atherosclerotic tissue on the performance of DREAMS 3 G remains unstudied. Accordingly, whether 3rd generation RMS provides better radial stability in presence of differing vessel wall characteristics compared to its predecessors is not clear. Intravascular optical coherence tomography (OCT) is an excellent tool to visualize the vascular lining and plaque components as it provides superior spatial resolution.

Against this background, the present analysis aims to assess the association between underlying plaque morphology and in‐scaffold late lumen loss (LLL) in patients treated with DREAMS 3 G through to 12‐months.

## 2 Methods

### 2.1 Study Population

The current study is a post‐hoc analysis of the BIOMAG‐I trial that includes patients who had intravascular OCT imaging acquired before and after the index procedure and qualitative coronary angiography (QCA) data available at 6‐ and 12‐months follow‐up. The BIOMAG‐I study (ClinicalTrials.gov: NCT04157153) was a multicentric, non‐randomized, single‐arm, first‐in‐human trial. Patients presenting with symptomatic coronary artery disease (stable/unstable angina, documented silent ischaemia, or non‐ST‐elevation myocardial infarction [NSTEMI]) and a maximum of two new single lesions in two separate coronary arteries were considered eligible for enrollment [4, 5]. The patients were considered ineligible in cases of ST‐elevation myocardial infarction (STEMI), unsuccessful pre‐dilatation, left main stenosis, or chronic total occlusion. The study protocol along with the full list of inclusion and exclusion criteria have been previously reported [4].

Ethics committees from each participating institution approved the study protocol and the study complied with the Declaration of Helsinki and Good Clinical Practice. All patients provided written informed consent before enrollment.

### 2.2 Study Device and Procedure

The backbone of DREAMS 3 G is composed of a resorbable magnesium alloy (called “BIOmag alloy,” consisting of 93.75% magnesium and 6.25% aluminum by weight) that is completely coated with bioresorbable poly L‐lactide acid, which incorporates sirolimus as the antiproliferative drug at a concentration of 140 μg/cm^2^. It contains two permanent X‐ray markers on its distal and proximal ends. Magnesium degrades completely within 12‐months to amorphous calcium phosphate through magnesium hydroxide and magnesium phosphate [3, 4]. The strut thickness varies depending on the scaffold diameters: 99 μm for 2.5 mm scaffold diameter, 117 μm for 3.0/3.5 mm scaffold diameter, and 147 μm for 4.0 mm scaffold diameter. The coating thickness ranges from 4 up to 15 μm. The stent was implanted in accordance with the “4 P” strategy [6, 7]. Implantation of a subsequent scaffold was allowed in case of incomplete lesion coverage or dissection, but with the condition to be placed end‐to‐end and without stent overlapping. All patients were recommended a dual antiplatelet therapy regimen for at least 6 months following the index procedure.

### 2.3 Data Acquisition

Coronary angiograms were acquired in two orthogonal views following intracoronary injection of 100−200 μg nitroglycerine, with matching projections taken before and after the index procedure. Intravascular OCT images were acquired at a pullback speed of 18 or 36 mm/s for the total length of the vessel segment. An automated injector was used (e.g., ACIST), with an infusion rate of > 4 mL/s (left coronary artery) or a rate of > 3 mL/s (right coronary artery) to attain a blood free environment for the OCT pullback.

### 2.4 Quantitative Coronary Angiography Analysis

All QCA data were sent to an independent core laboratory (MedStar Cardiovascular Research Network, Washington DC, USA). LLL at 6‐ and 12‐months was defined as the post‐procedural minimum lumen diameter (MLD) minus follow‐up MLD. In‐scaffold was defined as the longitudinal length from the proximal to the distal edge of the implanted scaffold. In‐segment was defined as the in‐scaffold length including 5 mm proximal and 5 mm distal to the stent edge.

### 2.5 Intravascular OCT Image Analysis

Intravascular OCT imaging data included in this study were analyzed offline by another independent imaging core laboratory (German Heart Center, Munich, Germany) using QIvus 3.1.18.0 software (Medis Medical Imaging Systems, Leiden NL). The qualitative and quantitative analyses were performed for every 1 mm along the entire segment of interest. A quality screening was performed for each pullback to confirm sufficient imaging quality. Patients were excluded from the analysis if: (1) OCT images were not available at any pre‐defined time points, (2) the image quality was insufficient for analysis, (3) less than 2/3 of the implanted scaffold length was available for analysis, (4) scaffolds of different thickness were implanted in the same lesion. By referring to proximal and distal edge markers along with anatomical landmarks (e.g., side branches, bifurcations, and calcifications) observed in the post‐procedural OCT data, target segments were identified in the pre‐procedural pullbacks.

For the qualitative analysis, a quadrant‐based approach was adopted for intimal characterization of the underlying plaque characteristics before the index procedure. Accordingly, each frame was subdivided into 4 quadrants of 90° and the plaque characteristics (normal, fibrous, calcific, or lipidic [8, 9]) were individually assessed for each one of them.

Quantitative analysis was performed for every 1 mm along the entire segment of interest with both stent and luminal cross‐sectional area measurements. The number of stent struts was recorded for each analyzed frame. Strut malapposition was considered present if the distance of the protruding strut between the luminal side of the strut and the luminal side of the vessel wall exceeded the sum of the implanted strut thickness (based on the diameter) and the maximum polymer thickness (15 μm). Struts identified at bifurcations or side‐branches were excluded. Post‐procedural edge dissections were defined as major if the visible edge dissection was > 60° of the circumference of the vessel or > 3 mm in length or as minor if any visible dissection was < 60° of the circumference of the vessel or < 3 mm in length [10].

### 2.6 Study Endpoints

For the current study, the main endpoint of interest was the degree of association between the pre‐procedural underlying plaque characteristics (normal, fibrous, calcific, or lipidic as per intravascular OCT imaging) and coronary angiogram based in‐scaffold LLL at 6‐ and 12‐months follow‐up. In addition, we investigated the potential impact of scaffold edge dissection and strut malapposition on in‐scaffold LLL.

### 2.7 Statistical Analysis

Categorical variables are reported as frequencies and percentages and were compared using the *χ* ^2^ or Fisher exact tests. Continuous data were checked for normality of distribution using the Wilk−Shapiro test and classified as parameters with normal or non‐parametric distribution. Continuous variables are expressed as mean ± standard deviation or median with interquartile range (IQR) and were compared using unpaired Student's *t*‐test or the non‐parametric Wilcoxon Kruskal−Wallis rank‐sum test. Proportion of plaque characteristics were calculated through dividing the sum of quadrants with corresponding plaque type by the total number of quadrants analyzed per patient. Non‐parametric Spearman's rank correlation was used to investigate the potential relationship in‐between underlying plaque characteristics, strut malapposition and in‐scaffold LLL at 6‐ and 12‐months. Hypothesis testing was performed at two tailed significance levels of 0.05. Statistical analysis was performed with JMP Pro, Version 16.0 (SAS Institute, Cary, NC, USA).

## 3 Results

**Figure 1:**
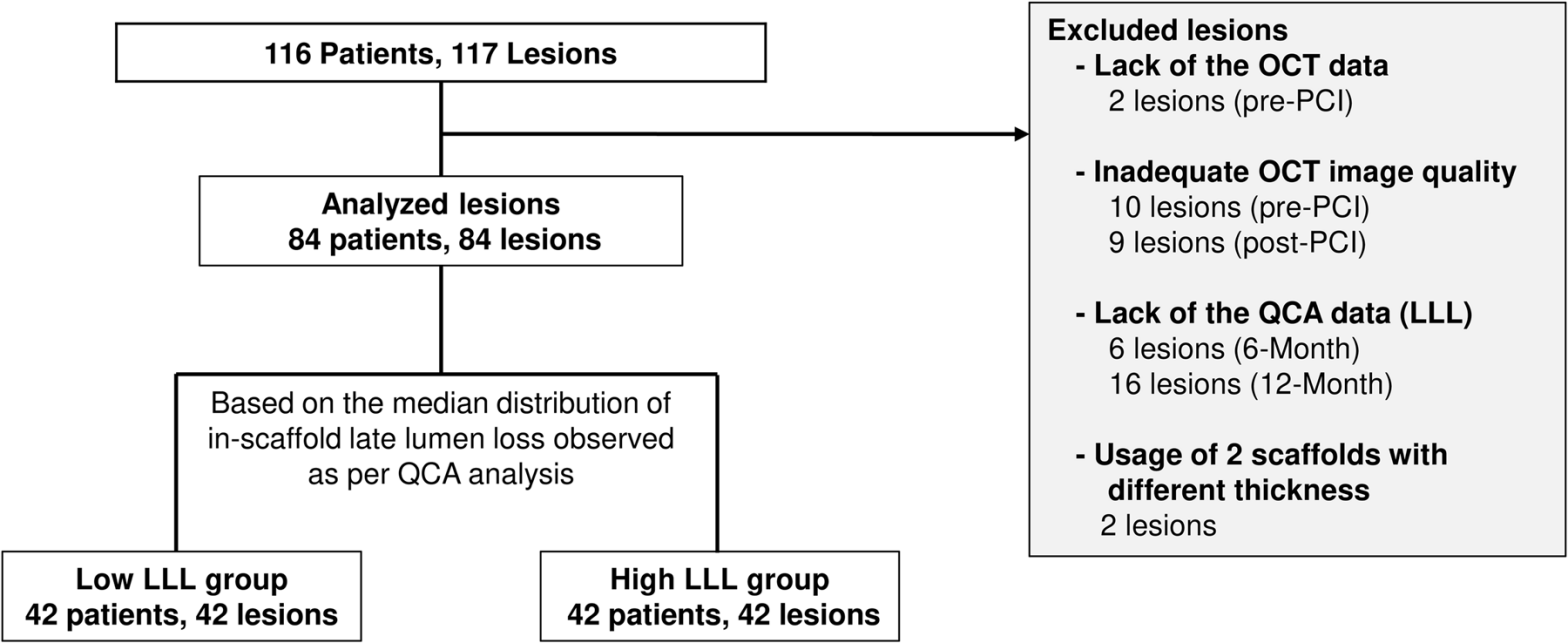
Study flowchart. LLL, late lumen loss; OCT, optical coherence tomography; QCA, quantitative coronary angiography.

Of 116 patients enrolled in the BIOMAG‐I trial, 84 patients (72.4%) and 84 lesions were included in the current analysis. Based on the median of distribution of in‐scaffold LLL at 12‐months (median value = 0.195), the study group was divided into low (*N* = 42; median value = 0.075) and high LLL (*N* = 42; median value = 0.385) groups to further investigate the influence of the underlying plaque characteristics. The study flow is shown in Figure 1.

### 3.1 Baseline Characteristics

Baseline patient and lesion characteristics for the overall cohort and two groups based on the median distribution of LLL are displayed in Table 1. The patients included in the current analysis tended to be older (61.2 ± 8.6 years) male (79.8%), more frequently hypertensive (75.0%) and with dyslipidaemia (65.5%). 15.5% of the patients presented with NSTEMI. A higher proportion of the treated lesions were in the left anterior descending artery (40.5%) with complex morphology according to AHA/ACC classification (46.4% and 31.0% were type B2 and C lesions, respectively). Low and high LLL groups were well‐balanced with regard to baseline characteristics.

**Table 1:** Patient and lesion characteristics based on the degree of in‐scaffold late lumen loss at 12‐months follow‐up.

Patient characteristics	All (*N* = 84)	Low LLL group (*N* = 42)	High LLL group (*N* = 42)	*p* value
Age (years)	61.2 ± 8.6	60.3 ± 8.2	62.1 ± 8.9	0.33
Sex				0.06
Male	67 (79.8)	37 (88.0)	30 (71.4)	
Female	17 (20.2)	5 (11.9)	12 (28.6)	
Previous myocardial infarction	27 (32.1)	16 (38.1)	11 (26.2)	0.24
Hypertension	63 (75.0)	30 (71.4)	33 (78.6)	0.45
Dyslipidemia	55 (65.5)	27 (64.3)	28 (66.7)	0.82
Diabetes mellitus	22 (26.2)	8 (19.0)	14 (33.3)	0.14
NSTEMI	13 (15.5)	6 (14.3)	7 (16.7)	0.76

Lesion characteristics	All (*N* = 84)	Low LLL group (*N* = 42)	High LLL group (*N* = 42)	*p* value
Lesion length (mm)	10.8 [8.2−14.7]	11.7 [8.58−15.70]	10.7 [7.55−14.33]	0.47
Target vessel				0.39
Left anterior descending artery	34 (40.5)	20 (47.6)	14 (33.3)	
Left circumflex artery	17 (20.2)	7 (16.7)	10 (23.8)	
Right coronary artery	33 (39.3)	15 (35.7)	18 (42.9)	
AHA/ACC classification				0.76
A	2 (2.4)	1 (2.4)	1 (2.4)	
B1	17 (20.2)	7 (16.7)	10 (23.8)	
B2	39 (46.4)	19 (45.2)	20 (47.6)	
C	26 (31.0)	15 (35.7)	11 (26.2)	

*Note:* Data are shown as mean ± standard deviation, median with interquartile range or counts (%).

Abbreviations: AHA/ACC, American heart association/American college of cardiology; LLL, late lumen loss; NSTEMI, non‐ST elevation acute coronary syndrome.

### 3.2 Procedural Characteristics

Pre‐ and post‐procedural characteristics as per intravascular OCT imaging for the overall cohort and two groups based on the median distribution of LLL are displayed in Table 2.

**Table 2:** Pre‐ and post‐procedural characteristics based on the degree of in‐scaffold late lumen loss at 12‐months follow‐up.

Pre‐procedural characteristics	All (*N* = 84)	Low LLL group (*N* = 42)	High LLL group (*N* = 42)	*p* value
Total number of analyzed frame	2072	1023	1049	
Analysis length	23.69 ± 5.97	23.39 ± 6.89	23.99 ± 4.95	0.65
Underlying plaque type^a^				
Proportion of normal quadrant	0.39 ± 0.21	0.39 ± 0.23	0.39 ± 0.19	0.94
Proportion of fibrous quadrant	0.43 ± 0.17	0.43 ± 0.20	0.44 ± 0.15	0.79
Proportion of calcium quadrant	0.06 ± 0.07	0.06 ± 0.07	0.06 ± 0.07	0.66^b^
Proportion of lipid quadrant	0.11 ± 0.14	0.12 ± 0.16	0.11 ± 0.11	0.84^b^

Post‐procedural characteristics	All (*N* = 84)	Low LLL group (*N* = 42)	High LLL group (*N* = 42)	*p* value
Total number of analyzed frame	2117	1043	1074	
Total number of analyzed struts	17,185	8241	8944	
Minimum lumen area (mm^2^)	6.68 [5.30−8.34]	6.92 [4.87−8.38]	6.49 [5.79−8.38]	0.83
Mean lumen area (mm^2^)	8.41 [6.78−10.53]	8.70 [6.57−10.60]	8.15 [6.94−10.52]	0.64
Average number of analyzed struts per pullback	204.58 ± 62.48	196.21 ± 69.19	212.95 ± 54.50	0.22
Analysis length	24.02 ± 6.84	23.48 ± 7.69	24.57 ± 5.92	0.38^b^
Strut malapposition	12.5 [7−22.75]	12.5 [7−22.75]	11 [5.75−23.25]	0.98
Edge dissection	28 (33.3)	14 (33.3)	14 (33.3)	1.00
None	56 (66.7)	28 (66.7)	28 (66.7)	
Minor	23 (27.4)	12 (28.6)	11 (26.2)	
Major	5 (5.9)	2 (4.8)	3 (7.1)	

*Note:* Data are shown as mean ± standard deviation, median with interquartile range or counts (%).

Abbreviation: LLL, late lumen loss.

a Proportion of plaque characteristics were calculated through dividing the sum of quadrants with corresponding plaque type by the total number of quadrants analyzed per patient.

^b^ The *p* value was calculated using the Wilcoxon test.

#### 3.2.1 Preprocedural Characteristics

**Figure 2:**
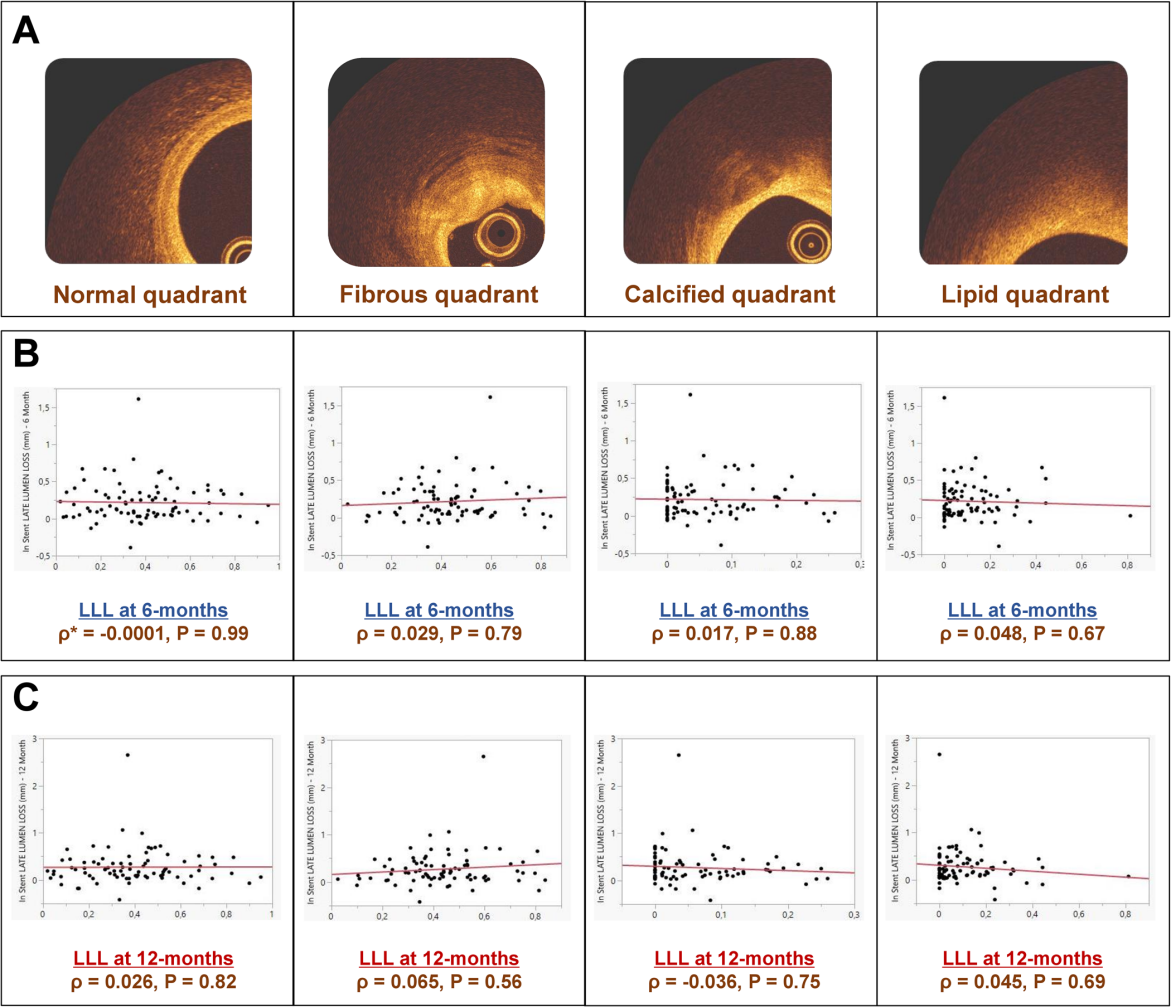
Correlation between underlying plaque characteristics and in‐scaffold LLL at 6‐ and 12‐months follow‐up. (A) Underlying plaque characteristics‐normal, fibrous, calcific, or lipidic‐ evaluated before the index procedure on a quadrant basis (4 quadrants of 90° per frame); (B) Correlation between the proportion of the corresponding plaque type and 6‐months in‐scaffold LLL; (C) Correlation between the proportion of the corresponding plaque type and 12‐months in‐scaffold LLL. *ρ denotes Spearman's rank correlation coefficient. LLL, late lumen loss. [Color figure can be viewed at wileyonlinelibrary.com]

Overall, 2072 frames were analyzed (1023 frames and 1049 frames in the low LLL and high LLL groups, respectively). There were no statistically significant differences between groups regarding the proportion of the underlying plaque type. A high proportion of the analyzed quadrants had fibrous plaques (low LLL vs. high LLL groups; 0.43 ± 0.20 vs. 0.44 ± 0.15, *p* = 0.79), followed by lipid (0.12 ± 0.16 vs. 0.11 ± 0.11, *p* = 0.84) and calcific plaques (0.06 ± 0.07 vs. 0.06 ± 0.07, *p* = 0.66). Correlation between the underlying plaque characteristics and in‐scaffold LLL at 6‐ and 12‐months follow‐up is displayed in Figure 2. There was no significant correlation between the underlying plaque characteristic and in‐scaffold LLL at 6‐months (*p* = 0.79 for fibrous, *p* = 0.88 for calcific, *p* = 0.67 for lipid lesions; Figure 2B). This trend was similar at 12 months follow‐up (*p* = 0.56 for fibrous, *p* = 0.75 for calcific, *p* = 0.69 for lipid lesions; Figure 2C).

#### 3.2.2 Post‐Procedural Characteristics

Overall, 2117 frames and 17,185 struts were analyzed (1043 frames, 8241 struts and 1074 frames, 8944 struts in the low LLL and high LLL groups, respectively). The average number of analyzed struts per pullback was 204.58 ± 62.48.

##### 3.2.2.1 Strut Malapposition

**Figure 3:**
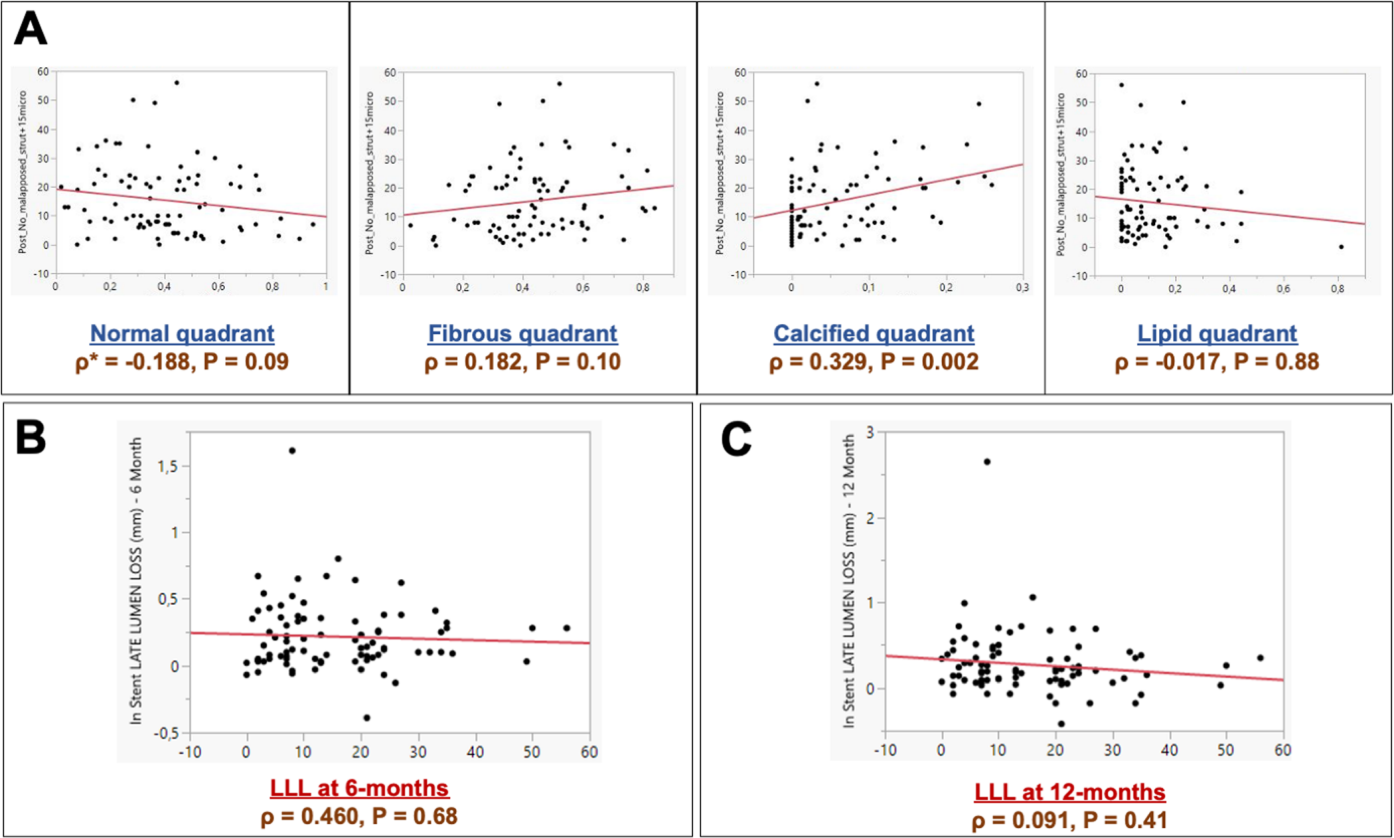
Correlation between strut malapposition, underlying plaque characteristics and in‐scaffold LLL at 6‐ and 12‐months follow‐up. (A) Correlation between strut malapposition and proportion of the corresponding plaque type (normal, fibrous, calcific, or lipidic); (B) Correlation between strut malapposition and 6‐months in‐scaffold LLL; (C) Correlation between strut malapposition and 12‐months in‐scaffold LLL. *ρ denotes Spearman's rank correlation coefficient. LLL, late lumen loss. [Color figure can be viewed at wileyonlinelibrary.com]

There were no statistically significant differences between groups regarding post‐procedural strut malapposition (low LLL vs. high LLL groups; 12.5 [7−22.75] vs. 11 [5.75−23.25], *p* = 0.98). Correlation between strut malapposition, underlying plaque characteristics and in‐scaffold LLL at 6‐ and 12‐months follow‐up is displayed in Figure 3. A significant association between the underlying calcific lesions and presence of acute strut malapposition (*p* = 0.002; Figure 3A) was observed. Still, this did not translate into a significant correlation between strut malapposition and in‐scaffold LLL at 6‐months (*p* = 0.68; Figure 3B) or 12‐months (*p* = 0.41; Figure 3C) follow‐up.

##### 3.2.2.2 Edge Dissection

**Figure 4:**
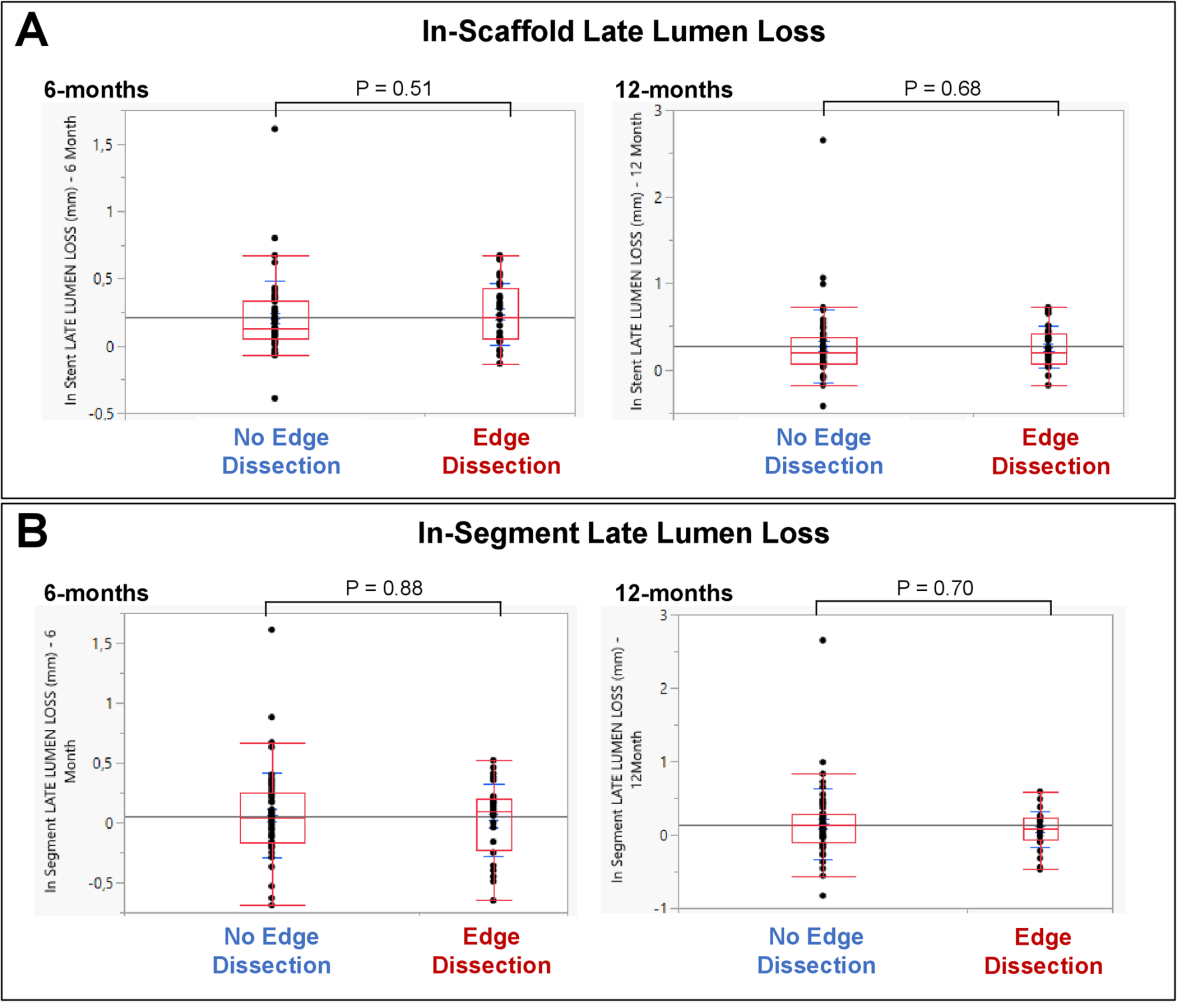
Degree of (A) in‐scaffold and (B) in‐segment late lumen loss based on the presence of stent edge dissection at 6‐ and 12‐months follow‐up. [Color figure can be viewed at wileyonlinelibrary.com]

Of patients included in this analysis, 33.3% had edge dissection. A higher proportion of these were minor dissections (low LLL vs. high LLL groups; 28.6% vs. 26.2%), followed by major dissections (low LLL vs. high LLL groups; 4.8% vs. 7.1%). To better understand the impact of edge dissection on LLL, we further assessed the extent of in‐scaffold and in‐segment LLL at 6‐ and 12‐months follow‐up based on the presence or absence of edge dissection. This is shown in Table 3 and Figure 4. The degree of in‐scaffold LLL did not significantly differ at 6‐months (No edge dissection vs. edge dissection; 0.13 [0.05−0.33] vs. 0.22 [0.05−0.43], *p* = 0.51) or 12‐months (0.20 [0.07‐0.38] vs. 0.20 [0.07−0.43], *p* = 0.68) follow‐up. Similarly, in‐segment LLL was comparable between groups at 6‐months (0.04 [−0.17 to 0.25] vs. 0.09 [−0.23 to 0.20], *p* = 0.88) and 12‐months (0.13 [−0.10 to 0.29] vs. 0.09 [−0.07 to 0.23], *p* = 0.70) follow‐up.

**Table 3:** In‐scaffold and in‐segment late lumen loss based on the presence of stent edge dissection at 6‐ and 12‐months follow‐up.

Characteristics	All (*N* = 84)	No edge dissection (*N* = 56)	Edge dissection (*N* = 28)	*p* value
In‐scaffold late lumen loss (mm)				
6 months	0.15 [0.05−0.35]	0.13 [0.05−0.33]	0.22 [0.05−0.43]	0.51
12 months	0.20 [0.07−0.39]	0.20 [0.07−0.38]	0.20 [0.07−0.43]	0.68
In‐segment late lumen loss (mm)				
6 months	0.06 [−0.17 to 0.22]	0.04 [−0.17 to 0.25]	0.09 [−0.23 to 0.20]	0.88
12 months	0.12 [−0.07 to 0.26]	0.13 [−0.10 to 0.29]	0.09 [−0.07 to 0.23]	0.70

*Note:* Data are shown as median with interquartile range.

## 4 Discussion

The key findings of this post hoc analysis of the BIOMAG‐I trial are as follows:

i. There was no significant correlation between the underlying plaque characteristics (fibrous, calcific, or lipid) and in‐scaffold LLL at 6‐ or 12‐months follow‐up.
ii. We observed a significant association between the underlying calcific plaques and acute strut malapposition. However, this did not translate into a significant correlation between strut malapposition and in‐scaffold LLL at longer‐term follow‐up.
iii. Presence of edge dissection had no influence on in‐scaffold or in‐segment LLL at 6‐ and 12‐months follow‐up.

Currently there is limited data regarding the potential impact of underlying plaque characteristics on in‐scaffold LLL at longer‐term follow‐up in patients treated with 3rd generation magnesium scaffold in a clinical trial.

Although the RMS technology indisputably showed improvements regarding the incidence of scaffold thrombosis [11], LLL still remains an important obstacle to overcome. Our analysis showed no significant correlation between the underlying plaque characteristics and in‐scaffold LLL at 6‐ or 12‐months follow‐up. Second generation Magmaris scaffold previously showed greater late recoil in presence of underlying fibrous plaque compared to calcified or lipidic plaque [2]. This suggests an improved radial force over an adequate duration with novel RMS compared to its predecessors. In line with our findings, Seguchi et al. [3] previously reported greater tensile strength as well as fewer strut discontinuities in a preclinical study comparing DREAMS 3 G and Magmaris scaffold, which are crucial factors for maintaining the radial force of a scaffold. At variance with our analysis, a recent study showed a significant association between fibrous tissue content at baseline and OCT‐derived minimum lumen area at 12‐months in patients enrolled in the BIOMAG‐I trial [12]. The differences in these findings may be explained by the methodologies applied to obtain the plaque‐based data. Garcia‐Garcia et al. [12] uses an AI based software to quantify the area of different plaque types and divided study population into 3 terciles. On the other hand, our study was based on a quadrant‐based frame‐to‐frame manual assessment of the underlying plaque characteristics with two separate groups based on the degree of LLL, aimed to align with the aforementioned analysis of the BIOSOLVE‐II trial [2].

Calcific plaques cause technical challenges for adequate lesion preparation, which in turn increase the risk of stent underexpansion as well as malapposition [13]. In the present study, while the “4 P” strategy was strictly adhered, a significant correlation was observed between the proportion of calcified quadrants and malapposed struts in patients treated with the DREAMS 3 G scaffold. This result suggests that, similar to drug‐eluting stents (DES), optimal implantation of RMS is also challenging in calcified plaques. It is important to note that there were no significant correlations observed between strut malapposition and in‐scaffold LLL at longer‐term follow‐up.

In patients undergoing PCI and treated with metallic stents, the impact of stent malapposition on long‐term adverse clinical outcomes remains unclear [14]. Nevertheless, acute stent malapposition is considered as a predictor of late and very late scaffold thrombosis following polymeric BRS implantation [15]. Sotomi et al. investigated the underlying causes of scaffold thrombosis following Absorb BVS implantation using intravascular imaging. Here, stent malapposition was the most frequently observed finding in patients with scaffold thrombosis [15]. At the 12‐month follow‐up of the BIOMAG‐I trial, there were no scaffold thrombosis observed [5]. Given the relatively low thrombogenicity of magnesium alloy in DREAMS 3 G owing to faster degradation [3], the impact of stent malapposition within RMS technology remains unclear. Further research is required to ascertain whether malapposition negatively impacts long‐term clinical outcomes.

Lastly, edge dissection is a common procedural complication observed with metallic stents, with previous reports showing an incidence of 5%−23% [16]. Owing to its high resolution, intravascular OCT is capable of detecting small edge dissections that might not be identified by intravascular ultrasound or coronary angiography. In a study by Chamié et al. using intravascular OCT imaging, the frequency of edge dissection was reported to be 39.1% [17]. Comparably, Radu et al. previously showed an incidence of 34% in patients treated with DES [18]. These findings are similar to the current analysis using the novel RMS, where we observed edge dissections in 33.3% of the study patients. Edge dissections following DES implantation have been reported to be associated with major adverse cardiac events (MACE), such as target lesion revascularization [19]. In this study, no significant correlation was observed between edge dissection and LLL. It is likely that the size of the present study population along with the follow‐up duration was insufficient to detect such correlation.

### 4.1 Limitations

This is a post‐hoc analysis of a non‐randomized study and as such it suffers the common potential limitations associated with not pre‐specified analyses. Accordingly, these results should be regarded as hypothesis‐generating. Intravascular imaging data were not available for all the patients, and selection bias may be present. BIOMAG‐I is a single‐arm study. As such, the present analysis does not involve a direct comparison of the DREAMS‐3G with metallic stents or polymeric BRSs. Furthermore, the possibility of type II error in statistics due to limited sample size is not to be excluded. Lastly, assessing plaque characteristics on a quadrant‐by‐quadrant may have caused inaccuracies in quantification, as plaques often exhibit multiple characteristics within the same quadrant.

## 5 Conclusions

The underlying plaque characteristics and the presence of edge dissection or strut malapposition had no significant impact on in‐scaffold LLL following DREAMS 3 G implantation up to 12 months. These results suggest better device performance with the novel RMS irrespective of the underlying plaque characteristics.

## Conflicts of Interest

The authors declare no conflicts of interest.

## Data Availability

The data underlying this article will be shared on reasonable request to the corresponding author.
